# Co-delivery of paclitaxel and cetuximab by nanodiamond enhances mitotic catastrophe and tumor inhibition

**DOI:** 10.1038/s41598-017-09983-8

**Published:** 2017-08-29

**Authors:** Yu-Wei Lin, Emmanuel Naveen Raj, Wei-Siang Liao, Johnson Lin, Kuang-Kai Liu, Ting-Hua Chen, Hsiao-Chun Cheng, Chi-Ching Wang, Lily Yi Li, Chinpiao Chen, Jui-I Chao

**Affiliations:** 1 Department and Institute of Biological Science and Technology, National Chiao Tung University, Hsinchu, 30068 Taiwan; 2 Institute of Molecular Medicine and Bioengineering, National Chiao Tung University, Hsinchu, 30068 Taiwan; 3 Hemato-Oncology Section, Department of Internal Medicine, Mackay Memorial Hospital, Taipei, 10449 Taiwan; 4 Department of Pharmaceutical Science, University of Toronto, Toronto, Ontario M5S 3M2 Canada; 5 Department of Chemistry, National Dong Hwa University, Hualien, 97401 Taiwan

## Abstract

The poor intracellular uptake and non-specific binding of anticancer drugs into cancer cells are the bottlenecks in cancer therapy. Nanocarrier platforms provide the opportunities to improve the drug efficacy. Here we show a carbon-based nanomaterial nanodiamond (ND) that carried paclitaxel (PTX), a microtubule inhibitor, and cetuximab (Cet), a specific monoclonal antibody against epidermal growth factor receptor (EGFR), inducing mitotic catastrophe and tumor inhibition in human colorectal cancer (CRC). ND-PTX blocked the mitotic progression, chromosomal separation, and induced apoptosis in the CRC cells; however, NDs did not induce these effects. Conjugation of ND-PTX with Cet (ND-PTX-Cet) was specifically binding to the EGFR-positive CRC cells and enhanced the mitotic catastrophe and apoptosis induction. Besides, ND-PTX-Cet markedly decreased tumor size in the xenograft EGFR-expressed human CRC tumors of nude mice. Moreover, ND-PTX-Cet induced the mitotic marker protein phospho-histone 3 (Ser10) and apoptotic protein active-caspase 3 for mitotic catastrophe and apoptosis. Taken together, this study demonstrated that the co-delivery of PTX and Cet by ND enhanced the effects of mitotic catastrophe and apoptosis *in vitro* and *in vivo*, which may be applied in the human CRC therapy.

## Introduction

Colorectal cancer (CRC) is one of the leading causes of morbidity and mortality worldwide^1, 2^. Specific molecular genes and proteins of CRC progression have been developed for therapeutic targets, including growth factor receptors, proliferation signaling, cell cycle progression and angiogenesis^3, 4^. Chemotherapeutic drugs and targeting antibodies can be used in treating CRC patients^5, 6^. However, the poor intracellular uptake, non-specific binding and limited circulation stability of those drugs declined its efficacy in cancer therapy.

Epidermal growth factor receptor (EGFR) of CRC cells is a crucial target for cancer therapy. Cetuximab (Cet) is a human/murine chimeric monoclonal antibody of EGFR with an immunoglobulin G1 Fc isotype^7^ that was treated in the metastatic CRC with the EGFR expression^8^. Cet can inhibit EGFR function by binding to the extracellular domain of EGFR and blocking ligand interaction^9^. Nonetheless, the drug resistance of Cet has been reported in clinical CRC patients^10, 11^.

Paclitaxel (PTX) is a microtubule inhibitor that can treat a variety of human cancers^12^. The mechanism of PTX is disturbing microtubule dynamics and inducing mitotic catastrophe^13–15^. Mitotic catastrophe is one of cell death types as a result of abnormal chromosome segregation by mitosis blockage^16–18^. The mitotic catastrophe in cancer cells induced by anticancer drugs contributed tumor suppression^16–18^. Moreover, PTX has drawbacks such as dose-limiting toxicity and drug resistance in cancer therapy.

The drug delivery systems by nanomaterials furnish the opportunity in improving the drug efficacy. Nanodiamond (ND) is a carbon-based nanomaterial without obvious cytotoxic effects in cellular^19–25^ and animals models^26–29^. The surface of NDs can be functionalized with chemicals or bio-molecules for various biomedical applications, including bio-labeling and drug delivery^30–39^. ND covalently linked with PTX was effective in drug delivery and tumor inhibition in human lung cancer^35, 40^. In this study, the co-delivery of Cet and PTX by ND enhances the mitotic catastrophe and tumor inhibition in the EGFR-expressed CRC.

## Results

### ND-PTX induces apoptosis in various human CRC cell lines

**Figure 1:**
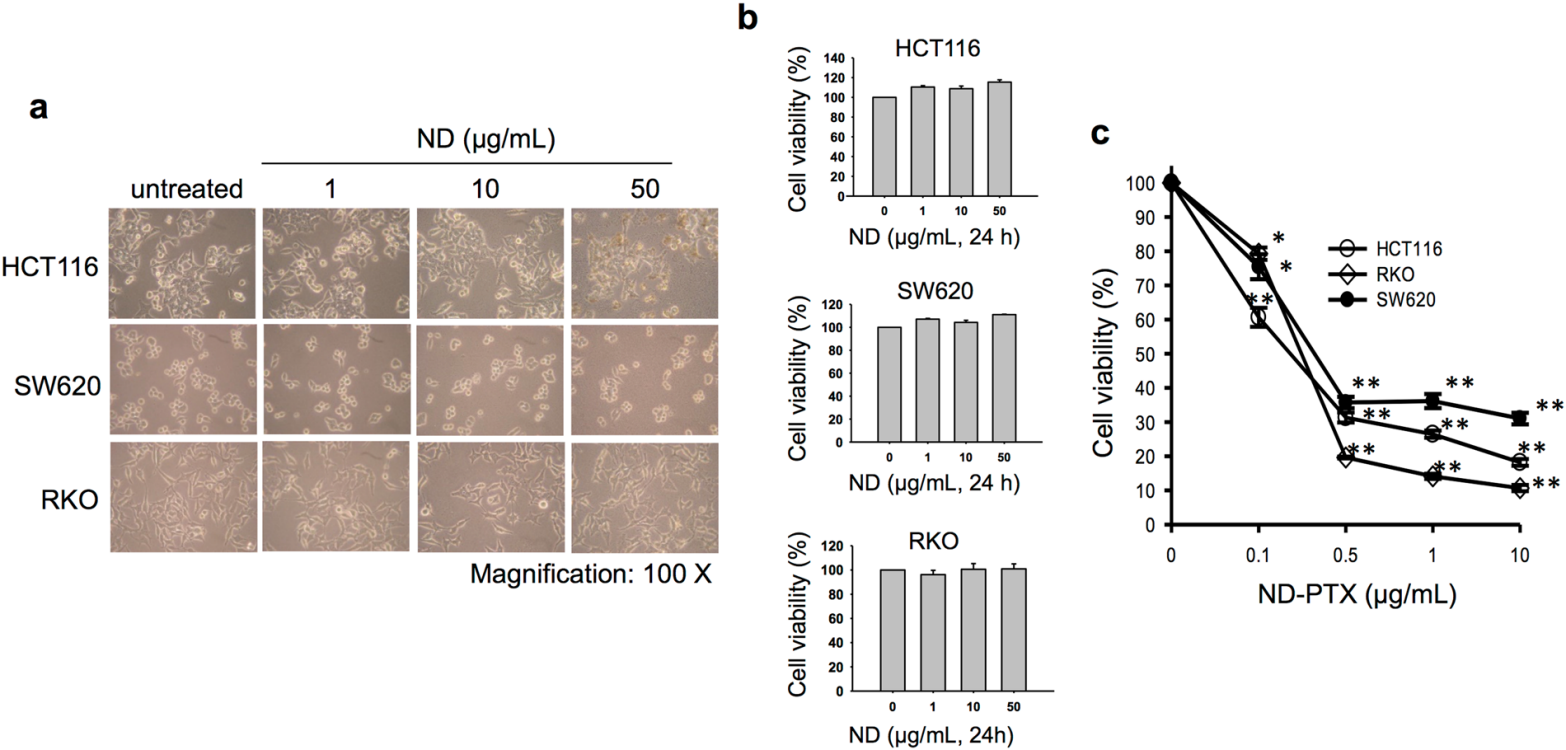
ND-PTX inhibits cell viability in various human CRC cells. (**a**) The human CRC cell lines (HCT116, SW620 and RKO) were left treated with or without NDs (1–50 μg/mL for 24 h). The cellular morphology was observed under a phase contrast microscope. (**b**) The cell viability after treatment with NDs was determined by MTT assays. (**c**) The cells were left treated with or without ND-PTX (0.1–10 μg/mL for 48 h). The cell viability was determined by MTT assays. The results were obtained from three separate experiments. The bars represent mean ± S.E. *p < 0.05 and **p < 0.01 indicate significant difference between untreated and ND-PTX treated samples.

Previous studies have indicated that NDs did not induce any obvious cytotoxic effects^19–25, 29^. We confirmed that NDs did not induce cytotoxic effects, including the cellular morphology and growth ability **(**Fig. 1a **)** and the cell viability **(**Fig. 1b **)** in various human CRC cell lines, HCT116, SW620 and RKO. We manipulated ND by covalently conjugated with PTX induced the blockage of lung tumor growth *in vitro* and *in vivo* ^35^. Nanocomplexes of PTX and NDs enhanced aqueous dispensability and drug retention in cancer cells^41^. Treatment with ND-PTX (0.1–10 μg/mL for 48 h) significantly reduced the cell viability in a concentration-dependent manner in HCT116, SW620 and RKO cells (Fig. 1c). Moreover, ND-PTX induced the cell death; however, ND alone did not induce these effects. (Supplementary Figure S1). We also compared the cell viability after treatment with PTX alone and ND-PTX, which contained similar concentrations to PTX, in the above cancer cell lines. Both PTX and ND-PTX could reduce the cell viability in the HCT116, SW620 and RKO cells (Supplementary Figure S2a and S2b). However, ND-PTX partially increased the reduction of cell viability by comparison with PTX alone. In addition, we confirmed that ND-PTX induced the cellular morphology of mitotic round up and apoptotic cells can be observed under a phase contrast microscope by time-lapse recording (Supplementary Figure S3 and Supporting video 1).

**Figure 2:**
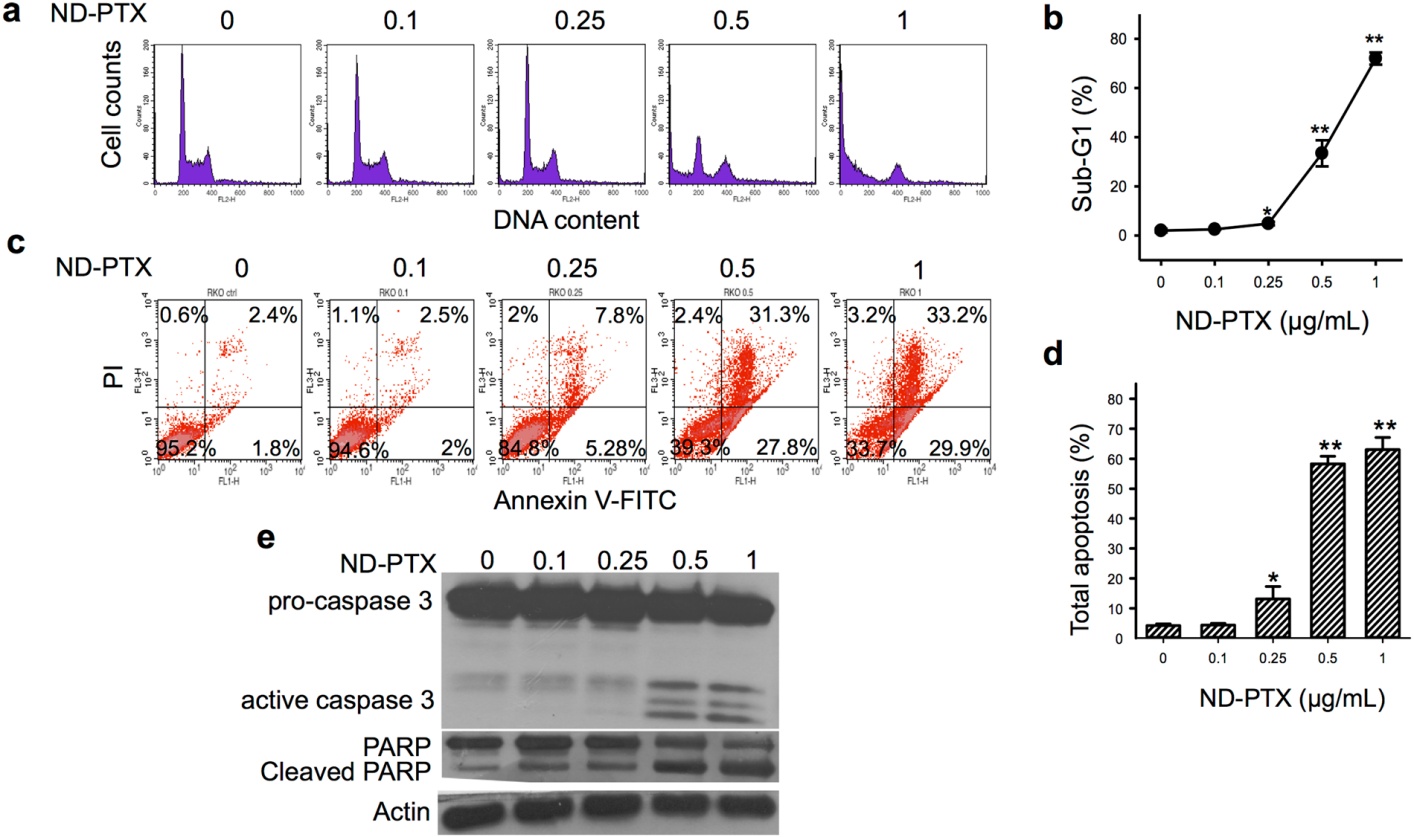
ND-PTX induces apoptosis through active caspase 3 and PARP cleavage in CRC cells. (**a**) RKO cells were left treated with or without 0.1–1 μg/mL ND-PTX for 48 h. The cellular sub-G1 fractions were analyzed by flow cytometry. (**b**) The sub-G1 fractions of cell population were quantified. (**c**) Apoptosis levels were determined by Annexin V-FITC and PI staining using flow cytometry. The population of Annexin V^+^/PI^−^ cells represents cells undergoing early apoptosis (lower right), whereas the fraction of Annexin V^+^/PI^+^ cells are those undergoing late apoptosis (upper right). (**d**) The percentage of total apoptosis (early and late apoptosis) cells was quantified. Results were obtained from three experiments and the bar represents the mean ± S.E. *p < 0.05 and **p < 0.01 indicate significant differences between untreated and ND-PTX samples. (**e**) RKO cells were left treated with or without 0.1–1 μg/mL ND-PTX for 48 h. At the end of treatment, the total protein extracts were collected and subjected to western blot. The representative western blot data were shown from one of three separate experiments with similar findings.

The uptake ability of ND-PTX in CRC cells was performed by flow cytometry. Treatment with ND-PTX (0.1–1 μg/mL for 48 h) elevated the fluorescence intensities of the cells in a concentration-dependent manner (Supplementary Figure S4). Meanwhile, ND-PTX significantly increased the sub-G_1_ fractions indicating apoptotic cell populations (Fig. 2a and b). We further confirmed ND-PTX-induced apoptosis levels by Annexin V-FITC and PI staining analysis. The population of Annexin V^+^/PI^−^ cells represents cells undergoing early apoptosis (lower right), whereas the fraction of Annexin V^+^/PI^+^ cells is undergoing late apoptosis (upper right). ND-PTX increased the percentages of early and late apoptosis in CRC cells (Fig. 2c). Quantification data show that ND-PTX induced total apoptosis levels in a concentration-dependent manner (Fig. 2d). The apoptotic proteins including the active caspase 3 and the cleaved PARP were induced by ND-PTX (Fig. 2e).

### ND-PTX induces mitotic catastrophe

**Figure 3:**
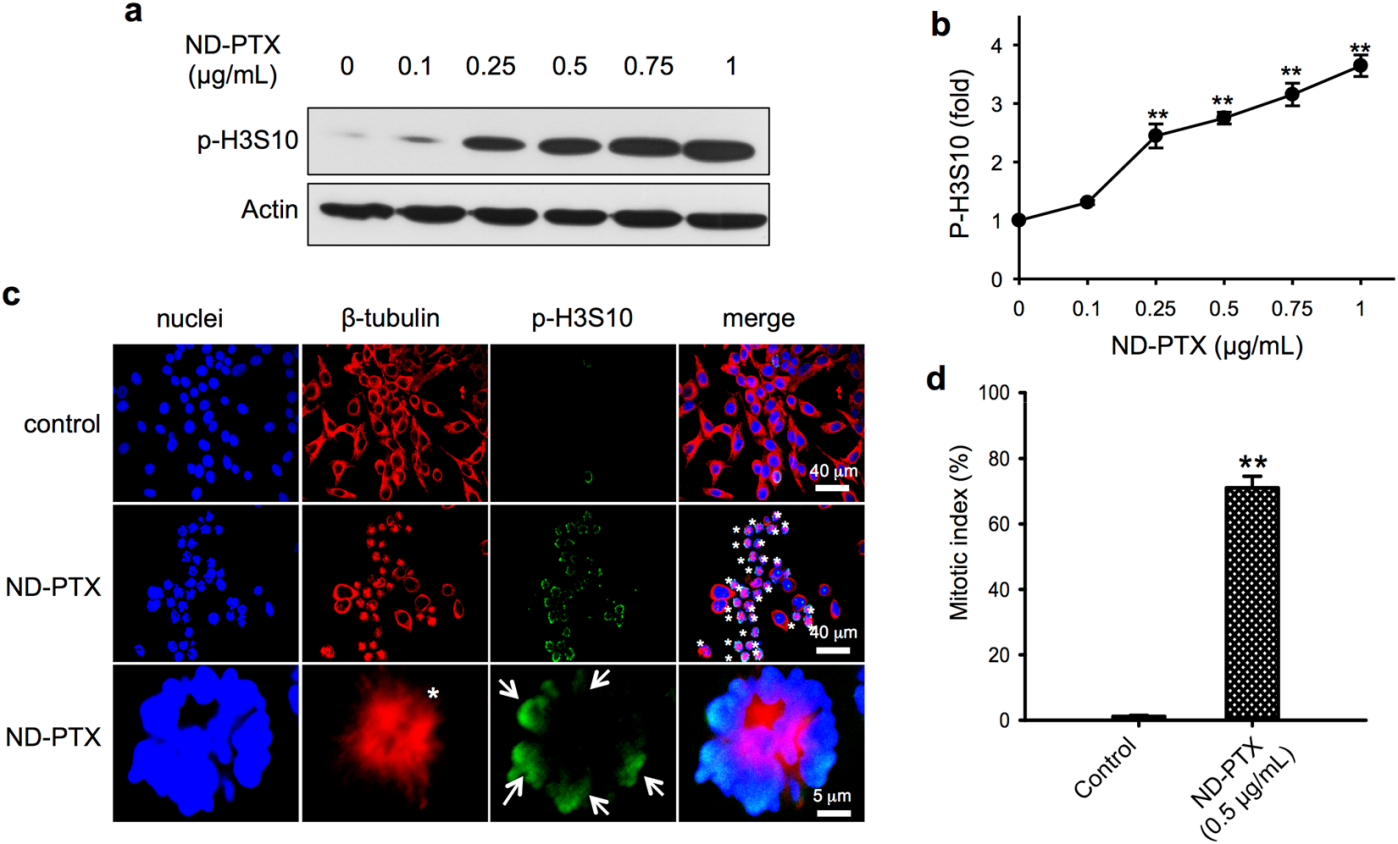
ND-PTX increases phospho-histone H3 proteins and mitotic index in human CRC cells. (**a**) RKO cells were left treated with or without ND-PTX (0.1–1 μg/mL for 24 h) as indicated. The total cellular protein extracts were subjected to western blot analysis using anti-p-H3S10 and anti-actin antibodies. Actin protein was used as an internal control. (**b**) The relative intensity of p-H3S10 was from western blot by semi-quantification. (**c**) RKO cells were treated with or without ND-PTX (0.5 μg/mL for 24 h). At the end of treatment, the cells were incubated with rabbit anti-p-H3S10 and then incubated with goat anti-rabbit IgG-Hylite 488. The β-tubulin and nuclei were stained with the Cy3-labeled mouse anti-β-tubulin and Hoechst 33258, respectively. (**d**) RKO cells were left treated with or without ND-PTX (0.5 μg/mL for 24 h). Mitotic index (the percentage of mitotic cell number/total cell number) was counted under a fluorescence microscope. Results were obtained from three experiments and the bar represents the mean ± S.E. **p < 0.01 indicates significant difference between control and ND-PTX treated samples.

**Figure 4:**
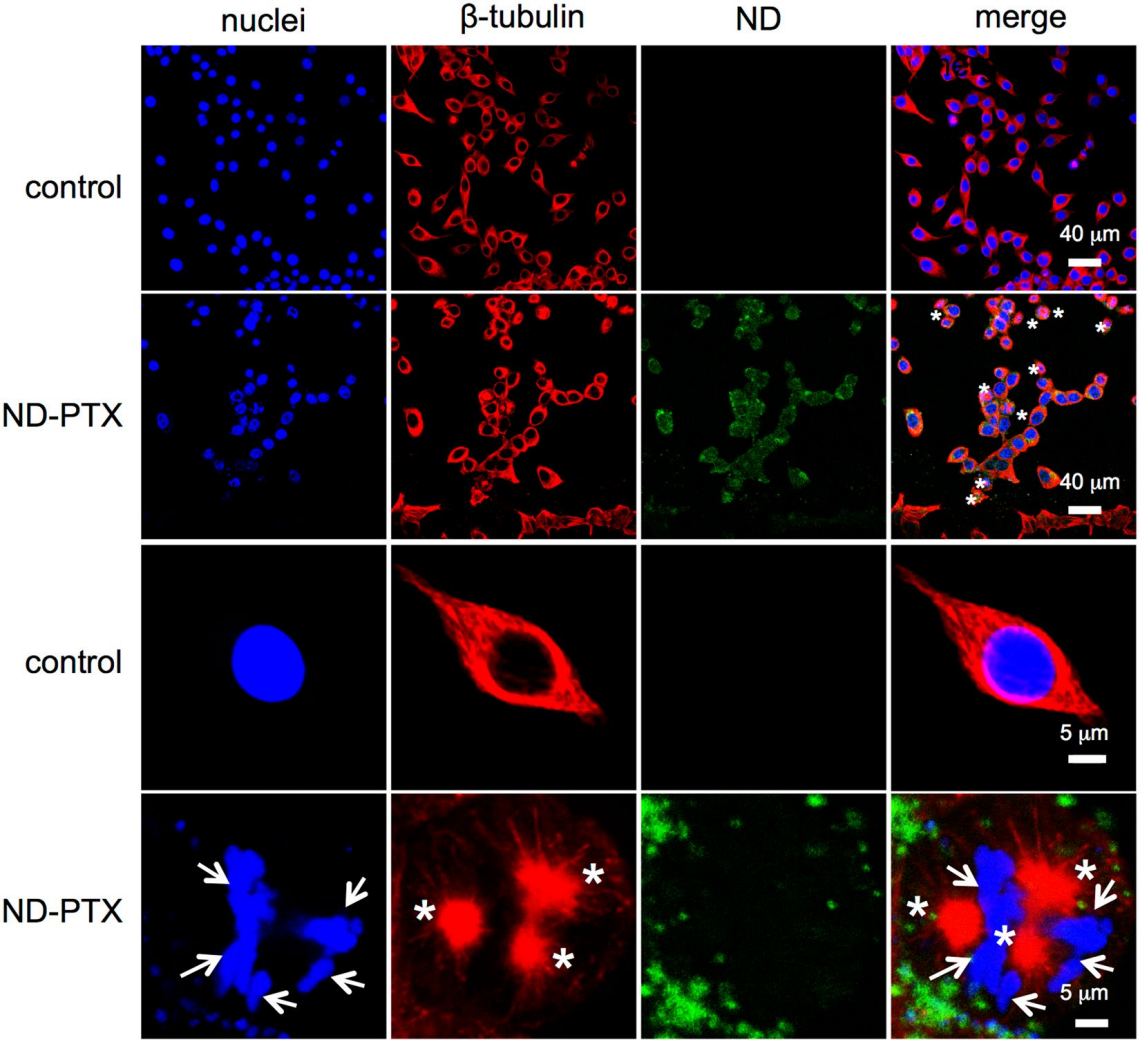
ND-PTX induces mitotic catastrophe in human CRC cells. RKO cells were left treated with or without ND-PTX and analyzed by confocal microscope. The green fluorescence from ND particles was excited by a wavelength of 488 nm and the emission was collected in the range 510–530 nm. Microtubule and nuclei exhibited red and blue color, respectively. The arrows indicate the abnormal microtubule formation. The stars indicate the abnormal chromosome segregation.

PTX disturbs microtubule dynamics by stabilizing the microtubule polymerization, which leads to mitotic catastrophe^13–15^. Histone phosphorylation is highly related to chromosome function during mitosis^42^. The phosphorylation of histone 3 (Ser10) (p-H3S10) is a hallmark of mitosis that begins during prophase, reaches maximum levels in metaphase^43^. As shown in Fig. 3a, ND-PTX (0.1–1 μg/mL for 24 h) increased the protein levels of p-H3S10 via a concentration-dependent manner in CRC cells. The quantified data showed that ND-PTX significantly increased the p-H3S10 proteins (Fig. 3b). Actin is an internal control protein that was not altered by ND-PTX treatment. To further confirm whether the ND-PTX-induce the increase of p-H3S10 proteins and microtubule inhibition, the cells were subjected to immunofluorescence staining and confocal microscopy. Treatment with ND-PTX (0.5 μg/mL for 24 h) increased the protein level of p-H3S10 (Fig. 3c, arrows). Moreover, ND-PTX blocked spindle formation and induced mitotic arrest cells (Fig. 3c, stars). ND-PTX significantly increased the mitotic index (percentage of counting mitotic cell number/total cell number) of ~75% (Fig. 3d). The abnormal spindle formation (stars) and chromosome segregation (arrows) were induced by ND-PTX (green) (Fig. 4).

### ND-PTX suppresses xenograft human CRC tumors in nude mice

**Figure 5:**
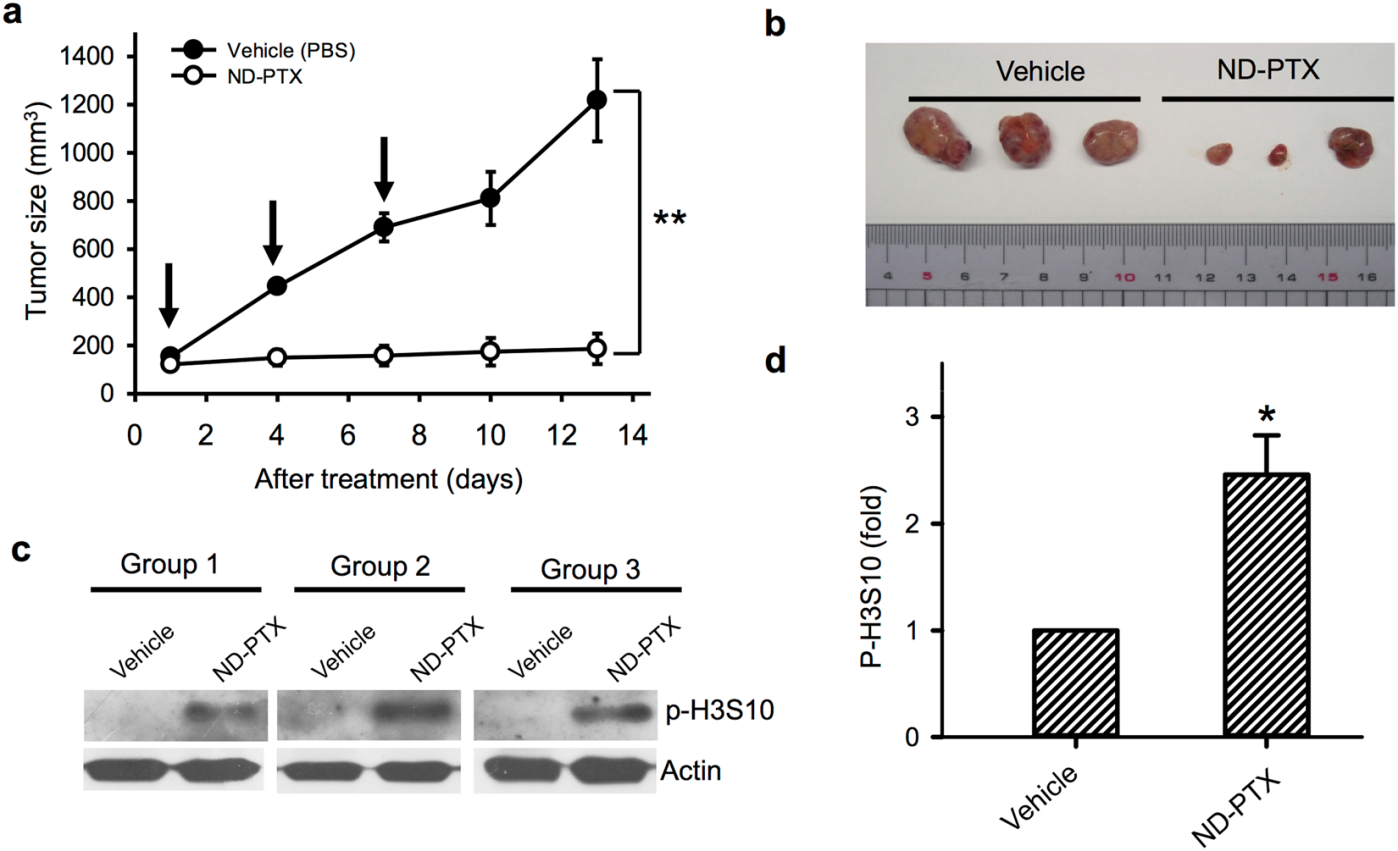
ND-PTX inhibits tumor size in the xenograft human CRC tumors of nude mice. (**a**) The four-week-old nude mice were subcutaneously injected with 2 × 10^6^ RKO cancer cells. After 10 days inoculation, the nude mice bearing xenografted colon tumors were treated with vehicle (PBS) or 50 mg/kg of ND-PTX three times for every 3 days (as the indicated by arrows). Each group contained three mice. The results were obtained from three mice and the bar represents the mean ± S.E. **p < 0.01, indicates significant difference between the control and ND-PTX treated samples. (**b**) The tumor size were compared from sacrificed mice between the vehicle and ND-PTX treated groups. (**c**) The xenograft tumor tissues from each group were homogenized and the total lysate were subjected to western blot analysis using specific antibodies for p-H3S10 and actin. (**d**) The relative protein intensity of p-H3S10 from western analysis was analyzed by semi-quantification. The results were obtained from three experiments and the bar represents the mean ± S.E. *p < 0.05 indicates significant difference between the control and ND-PTX treated samples.

To evaluate the antitumor ability of ND-PTX in CRC *in vivo*, the effect of ND-PTX on the xenografted human CRC tumors in nude mice was examined. RKO cells were subcutaneously injected into the nude mice for 10 days and then treated with 50 mg/kg of ND-PTX three times for every 3 days. Figure 5a shows that the tumor volumes were significantly reduced in the ND-PTX treated groups by comparison with the control groups. In the control groups, the average tumor volume was 1218.19 mm^3^ (Fig. 5b); however, the average tumor volume in the ND-PTX treated groups was reduced to 186.23 mm^3^ (Fig. 5b). Besides, the tumor tissues sacrificed from mice were examined the p-H3S10 protein levels by western blot. ND-PTX significantly increased the protein levels of p-H3S10 in the xenografted tumors (Fig. 5c and d). Actin is an internal control protein that did not alter following treatment with ND-PTX.

### ND-PTX-Cet binds selectively to the EGFR-expressed CRC cells

**Figure 6:**
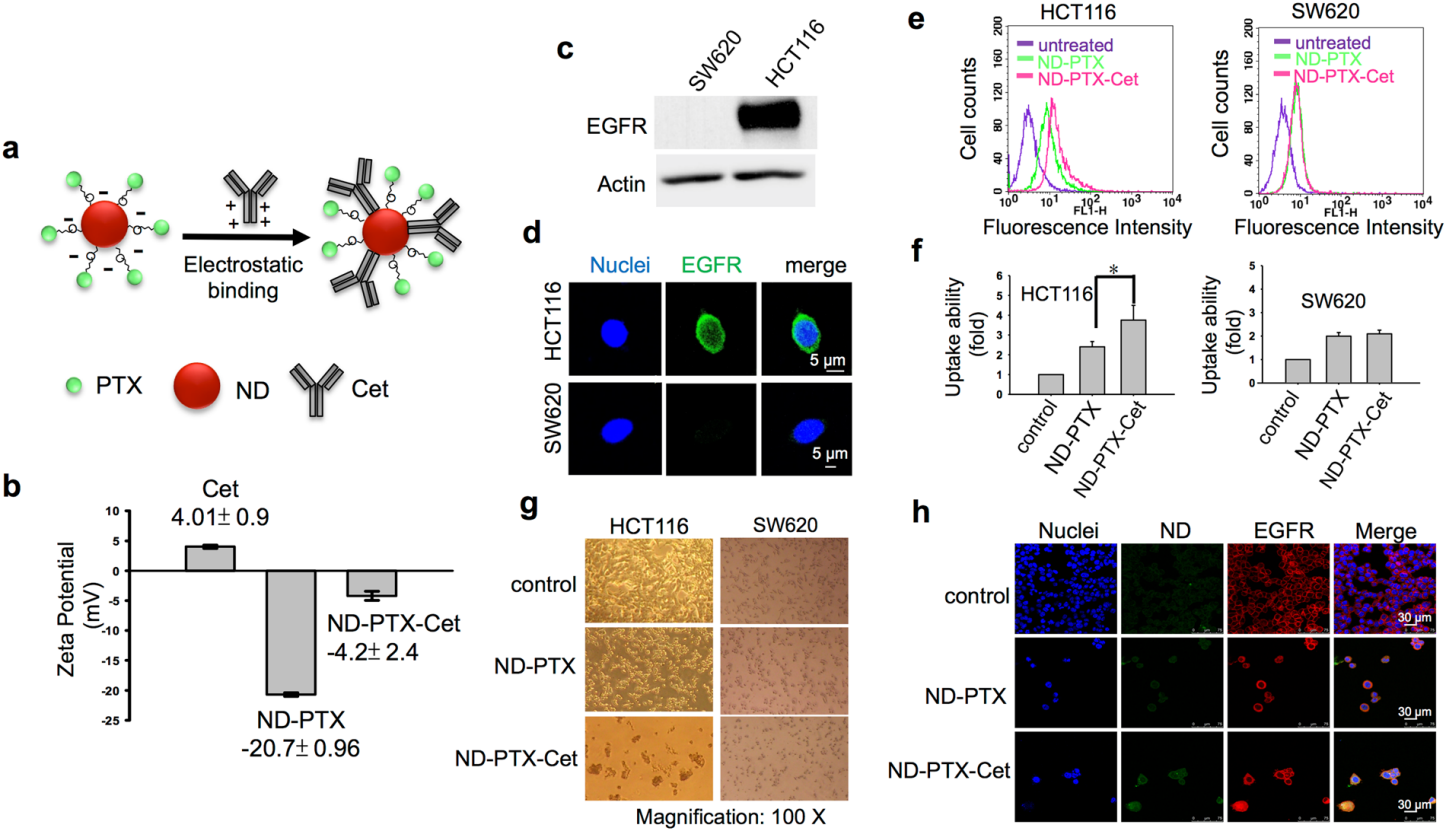
ND-PTX-Cet enhances the cellular uptake ability and apoptosis in the EGFR-expressed CRC cells. (**a**) The conjugation model of ND-PTX and Cet. (**b**) The surface charges of Cet, ND-PTX and ND-PTX-Cet were measured by Zeta potential instrument. (**c**) The EGFR protein levels in SW620 and HCT116 cells were analyzed by western blot. (**d**) The green color shows the location of EGFR proteins. Nuclei were stained with the Hoechst 33258 (blue). (**e**) After treatment with or without 10 μg/mL of ND-PTX or ND-PTX-Cet for 24 h, the cells were analyzed by flow cytometry. (**f**) The fluorescence intensity of ND-PTX in cells was quantified. The bar represents the mean ± S.E. *p < 0.05 indicates significant difference between ND-PTX and ND-PTX-Cet. (**g**) The cells were left treated with or without 1 μg/mL of ND-PTX or ND-PTX-Cet for 48 h. The cell morphology was observed under a live-cell imaging microscope. (**h**) The green color shows the location of ND particles. EGFR (red) and nuclei (blue) were exhibited.

**Figure 7:**
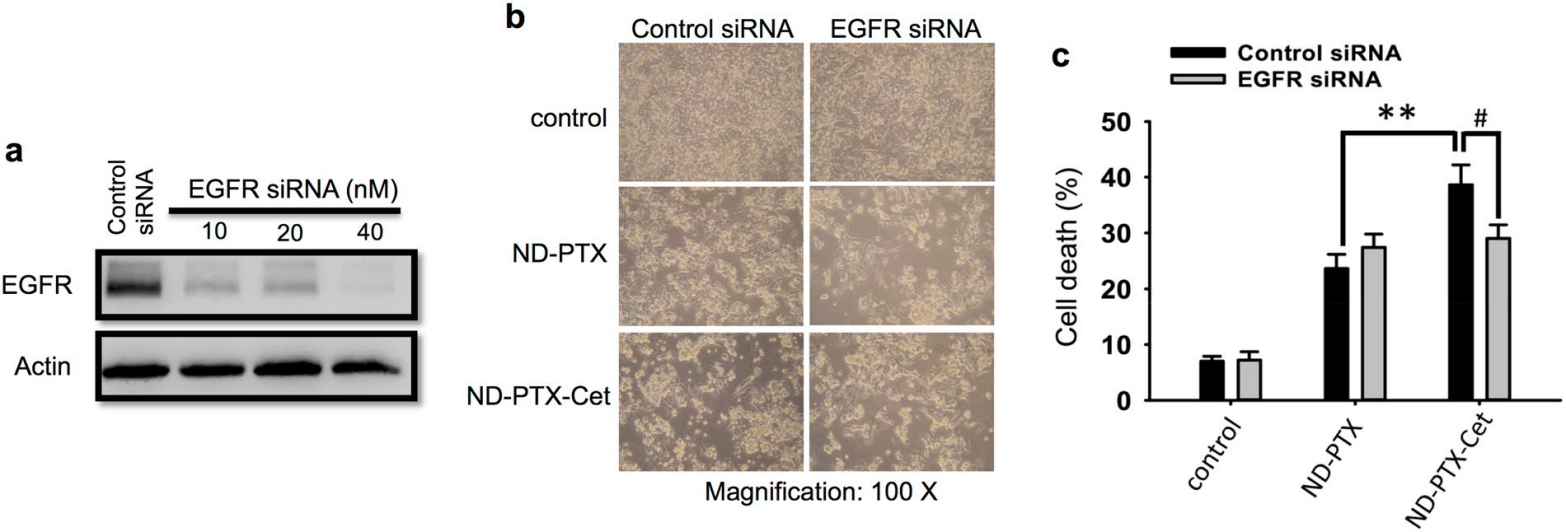
The knockdown of EGFR expression reduces the cell death effect of ND-PTX-Cet in human CRC cells. (**a**) HCT116 cells were plated at a density of 7 × 10^5^ cells per 60-mm Petri dish in complete medium for 18–20 h. The cells were transfected with or without EGFR siRNA (10–40 nM for 48 h) in serum-free medium for 6 h at 37 °C. Then, the equal amount with 20% FBS medium was added without removing the transfection mixture, and incubation proceeded for an additional 48 h. At the end of treatment, the total protein extracts were prepared for western blot analyses using specific antibodies, including rabbit anti-EGFR and mouse anti-actin. (**b**) HCT116 cells were transfected with or without EGFR siRNA (10 nM) for 48 h and then the cells were treated with or without ND-PTX (1 μg/mL) and ND-PTX-Cet (1 μg/mL) for 48 h. The treated cells were immediately observed under a live-cell imaging microscope. (**c**) At the end of treatment, the total cell numbers were collected and the percentages of cell death were determined by automatic cell counter. The bar represents the mean ± S.E. ^#^ *p* < 0.05 indicates significant difference between the control and EGFR siRNA by treatment with ND-PTX-Cet. **p < 0.01 indicates significant difference between ND-PTX and ND-PTX-Cet by transfection with control siRNA.

Figure 6a shows a model of the conjugation of ND-PTX-Cet. The surface charge of Cet, ND-PTX and ND-PTX-Cet was analyzed by Zeta potential instrument. The surface charge of Cet and ND-PTX was 4.01 ± 0.9 and −20.7 ± 0.96, respectively. The average surface charge was −4.2 ± 2.4 when ND-PTX bound with Cet through electrostatic interaction (Fig. 6b). HCT116 cells highly expressed EGFR proteins while SW620 did not express EGFR proteins analyzed by western blot and confocal microscopy. (Fig. 6c and d). Treatment with ND-PTX-Cet enhanced the cellular uptake ability in the HCT116 cells but not in the SW620 cells (Fig. 6e and f). ND-PTX-Cet enhanced the cell death in HCT116 cells but had no significant effect in the SW620 cells (Fig. 6g). Besides, ND-PTX-Cet (green) can selectively bind to EGFR (Fig. 6h). Moreover, the EGFR protein expression of HCT116 cells was reduced by transfection with EGFR siRNA (Fig. 7a). The depletion of EGFR proteins by EGFR siRNA significantly reduced cell death by comparing with EGFR siRNA in the ND-PTX-Cet treated cells (Fig. 7b and c).

### ND-PTX-Cet inhibits the xenografted human drug-resistant tumors in nude mice

**Figure 8:**
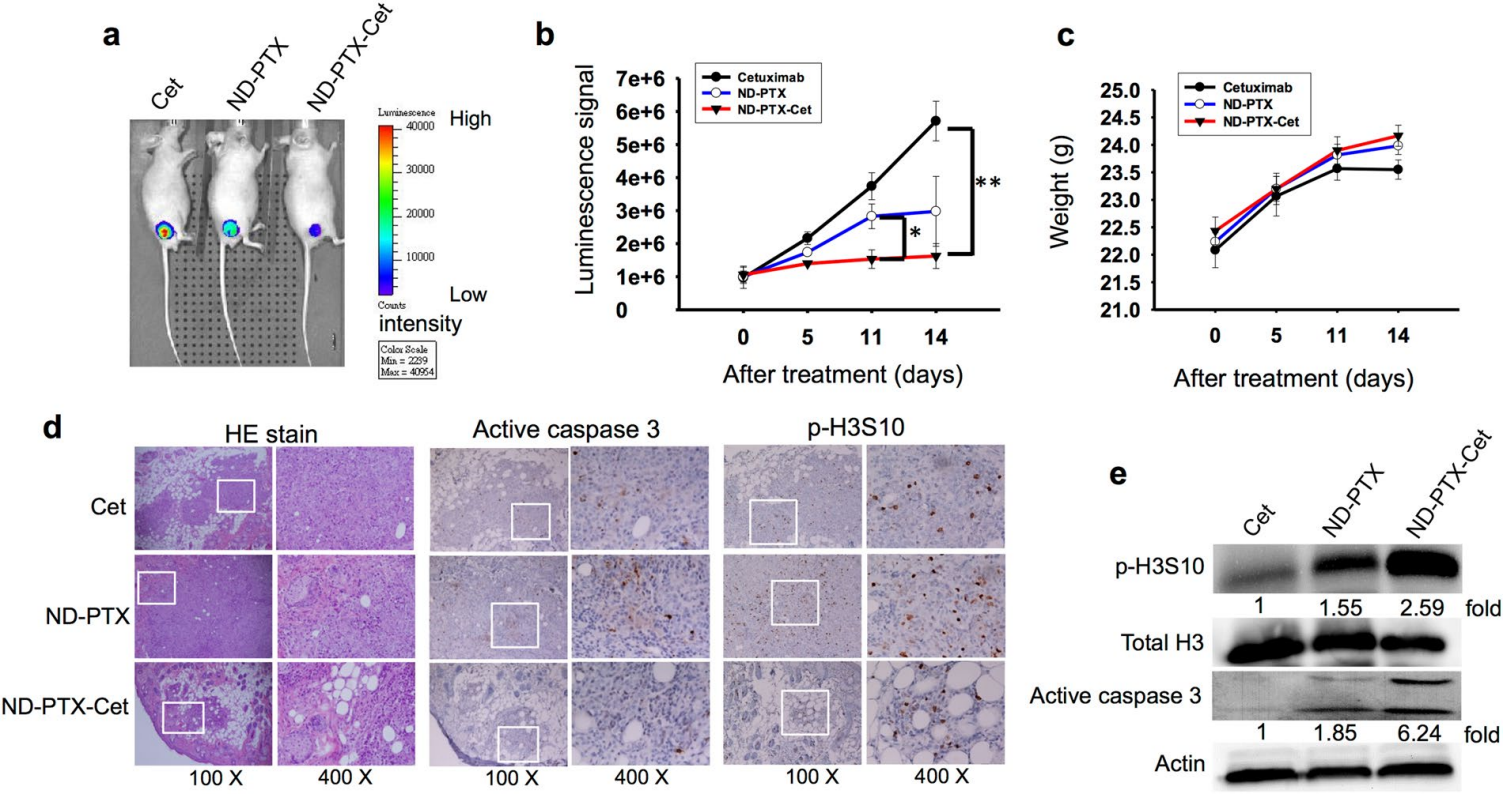
ND-PTX-Cet enhances the apoptosis induction and tumor size reduction in the xenograft human CRC tumors in nude mice. (**a**) The nude mice were subcutaneously injected with 2 × 10^6^ HCT116-luc2 cells. After inoculation, the mice bearing human CRC tumors were treated with or without 20 mg/kg of Cet, ND-PTX or of ND-PTX-Cet. The luminescence intensities of HCT116-luc2 xenograft tumors were observed by IVIS system. (**b**) The quantified data show during 14 days of drug administration. Values shown are means ± S.E. for the indicated group sizes. *p < 0.05 and **p < 0.01 indicate significant differences between control and treated mice. (**c**) Comparison of the body weights of drug-injected mice at a time period of 14 days. (**d**) The tumor tissues were from sacrificed mice and analyzed by hematoxylin and eosin (HE) staining. The p-H3S10 and active caspase 3 proteins were also examined by IHC staining. Representative photographs were by IHC staining the active caspase 3 and p-Histone H3 (Ser10) proteins in the tumor sections. (**e**) The xenograft tumor tissues from each group were homogenized and the total lysate were subjected to western blot using specific antibodies for p-H3S10, total H3, caspase 3 and actin. The relative intensity (fold) of proteins was semi-quantified.

To compare the antitumor activity between ND-PTX and ND-PTX-Cet, the HCT116-luc2 cells were subcutaneously inoculated into the right flank for 7 to 10 days and then treated with 20 mg/kg of Cet, 20 mg/kg of ND-PTX and ND-PTX-Cet three times for every 3 days. The luminescence signal of CRC tumors was reduced in the ND-PTX-Cet treatment groups by comparing to the control and ND-PTX treatment groups **(**Fig. 8a and b). However, the body weight of control group and drug-treated mice were not altered (Fig. 8c). The histological analysis by H&E staining revealed a widespread cystic space inside tumor due to tumor destruction by ND-PTX-Cet treatment (Fig. 8d). ND-PTX-Cet significantly increased the protein levels of p-H3S10 and the active forms of caspase 3 in the xenografted colon tumors by comparing with control or ND-PTX groups (Fig. 8d and e).

## Discussion

The use of nanocarriers as drug delivery systems provide the opportunities to improve the efficacy of cancer therapy^44–46^. Various chemotherapeutic drugs can be linked to NDs^35, 40, 47–50^. ND-conjugated PTX has shown great promise in drug delivery and tumor blockage^35, 40, 41^. In this study, ND-PTX effectively inhibited CRC cell growth in cellular and animal models. Furthermore, the co-delivery of PTX and Cet by ND enhanced the mitotic catastrophe and tumor inhibition in the drug-resistance of CRC *in vitro* and *in vivo*.

The resistance to chemotherapy is acquired after treatment in cancer patients. ND is a promising nanomaterial for delivering chemotherapeutic drugs. ND-carried drugs could overcome drug efflux and multi-drug resistance to block tumors^51, 52^. ND delivered drugs to cancer cells primarily by endocytosis, which can bypass the multiple drug resistance (MDR) proteins. NDs can be taken by cancer cells through macropinocytosis and clathrin-mediated endocytosis^20^. ND conjugated with daunorubicin has been reported to be better performance in drug resistant leukemia cells^52^. ND as drug delivery system may improve drug retention and treatment efficacy in those chemoresistance tumors. Besides, ND-PTX-Cet exerted anticancer ability in drug-resistant K-Ras mutation of HCT116 CRC cells. We suggest that ND-PTX-Cet can overcome several drug-resistant forms in human cancers.

The phosphorylation of histone 3 (Ser10) (p-H3S10) is a hallmark of mitosis that begins during prophase, reaches maximum levels in metaphase^43^. Mitotic catastrophe has been accompanied by apoptosis induction^17^. The levels of p-H3S10 plays pivotal role in regulation of apoptosis and mitotic catastrophe^53–56^. PTX is a microtubule inhibitor by stabilizing the microtubule polymerization, which leads to mitotic catastrophe^13–15^. ND displayed fluorescence property without photobleaching^19, 24, 26, 57^. The intracellular location and uptake ability of ND-PTX can be detected by confocal microscope or flow cytometer. Previously, we showed that ND delivered into lysosomes through the selective autophagy pathway^57^. The uptake ability of ND-PTX is correlated to the anticancer activity of PTX in inducing mitotic catastrophe. ND-PTX increased the p-H3S10 proteins in the CRC cells and xenografted human tumor tissues in nude mice. Moreover, ND-PTX induced the apoptotic caspase 3 activation and the cleavage of PARP proteins. PARP is a key substrate for caspase 3 that was cleaved when cells undergo apoptosis^58^. These results demonstrate that ND-PTX can deliver into cancer cells to induce the mitotic catastrophe and apoptosis in human CRC.

Cet is a monoclonal antibody of EGFR that can provide metastatic CRC therapy^7, 8^. Cet can block EGFR function by binding to the extracellular domain of EGFR^9^. Conjugation of ND-PTX with Cet could specifically bind on the EGFR-positive CRC cells to enhance the mitotic blockage and apoptosis induction. ND-PTX-Cet markedly decreased tumor growth in the human EGFR-expressed CRC xenograft tumors of nude mice. Moreover, ND-PTX-Cet induced the persisted expression of p-H3S10 and active caspase 3 proteins for the mitotic catastrophe and apoptosis induction *in vitro* and *in vivo*.

In conclusion, we demonstrated that the new formulation of ND-PTX-Cet enhanced mitotic catastrophe, apoptosis induction and tumor inhibition. ND-PTX-Cet may develop a novel nanocomposite for the human CRC therapy.

## Methods

### Reagents and antibodies

Propidium iodide (PI), 3-(4,5-dimethyl-thiazol-2-yl) 2,5-diphenyl tetrazolium bromide (MTT), Hoechst 33258, and the Cy3-labeled mouse anti-β-tubulin (c-4585) were purchased from Sigma Chemical Co. (St Louis, MO, USA). Anti-poly(ADP-ribose) polymerase (PARP) antibody was purchased from Cell Signaling Technology Inc. (Beverly, MA). Anti-caspase 3 antibody was purchased from BioVision (BioVision, Inc., Mountain View, CA). Anti-actin (I-19) antibody, goat anti-rabbit IgG horseradish peroxidase, goat anti-mouse IgG horseradish peroxidase and EGFR siRNA were purchased from Santa Cruz Biotechnology, Inc. (Santa Cruz, CA). Anti-phospho-histone H3 (Ser10) (p-H3S10) was purchased from Cell Signaling Technology, Inc. (Beverly, MA, USA).

### Preparation of ND-PTX and ND-PTX-Cet

Paclitaxel was purchased from Tokyo Chemical Industry Co. (Ltd. Japan). Powdered ND particles with diameters of 3–5 nm were purchased from Nanostructured and Amorphous Materials Inc. (Houston, TX, USA). Initial chemical treatment of ND powders by carboxylation was carried out according to standard procedure^35^. Briefly, the ND particles were stirred in a 3:1 (v/v) mixture of concentrated HCl and HNO_3_ at room temperature for three days, then diluted with distillated deionized H_2_O (DDW) and separated by centrifugation at 900 rpm. After centrifugation, the pellets were extensively rinsed with DDW three times. The chemical synthetic procedure for the conjugation of ND and paclitaxel (PTX) was synthesized as described previously^35^. Cetuximab (Cet) was purchased from Merck Serono (Darmstadt, DE). For conjugation of ND-PTX and Cet, 10 µg of ND-PTX was incubated with 2.5 µg of Cet to form the ND-PTX-Cet complexes in DDW at 4 °C for 15 min. To avoid the unconjugated Cet, the ND-PTX-Cet complexes were centrifuged at 10000 rpm for 10 min at 4 °C in DDW. Thereafter, the pellets of ND-PTX-Cet complexes were collected and washed with DDW twice. For cellular and animal experiments, the concentrations of ND, ND-PTX and ND-PTX-Cet were freshly prepared with DDW for treatment in laminar flow.

### Cell lines and cell culture

RKO (ATCC number: CRL-2677) was a colorectal carcinoma cell line that expressed wild type EGFR and K-Ras. HCT116 (ATCC number: CCL-247) was a colorectal carcinoma cell line that expressed wild type EGFR but carried K-Ras mutation. SW620 was a colorectal carcinoma cell line that did not express the EGFR proteins. RKO cells were cultured in DMEM medium (Gibco, Life Technologies, Grand Island, NY, USA). HCT116 cells were maintained in McCoy’s 5 A medium (Gibco, Life Technologies). SW620 were maintained in L-15 medium (Gibco, Life Technologies). The complete medium was supplemented with 10% fetal bovine serum (FBS), 100 units/mL penicillin, 100 μg/mL streptomycin and sodium bicarbonate. These cells were maintained at 37 °C and 5% CO_2_ in a humidified incubator (310/Thermo, Forma Scientific, Inc., Marietta, OH).

### Cell viability assays

Briefly, the cells were plated in 96-well plates at a density of 1 × 10^4^ cells/well for 16–20 h. Then the cells were treated with ND or ND-conjugates in complete medium. At the end of treatment, the medium was replaced and the cells were incubated with 0.5 mg/mL of MTT in complete DMEM medium for 4 h. The viable cells converted MTT to formazan that generated a blue-purple color when dissolved in DMSO. The intensity of formazan was measured at 565 nm using a plate reader (VERSAmax, Molecular Dynamics, Sunnyvale, CA). The relative percentage of cell viability was calculated by dividing the absorbance of treated cells by that of the control in each experiment.

### Cell death assays

The cells were plated at a density of 1 × 10^6^ cells per 60-mm Petri dish in complete medium for 16–20 h. Thereafter, the cells were treated with ND or ND-conjugates. After treatment, the cells were washed with PBS and re-cultured in fresh medium. Finally, the samples were mixed with the Accustain solution, which contained lysis solution and PI. The cell death number by PI staining was subjected to an automatic cell counter (ADAM-MC, NESMU-AMC-001E, Digital Bio, Korean).

### Uptake ability of ND-conjugates in the cells by flow cytometry

The cells were plated at a density of 7 × 10^5^ cells per 60-mm Petri dish in complete medium for 16–20 h. After treatment with ND-conjugates, the cells were collected and fixed with ice-cold 70% ethanol overnight at −20 °C. The samples were analyzed by a flow cytometer (FACSCalibur, BD Biosciences, San Jose, CA). A minimum of ten-thousand cells was analyzed. The fluorescence of NDs was excited with wavelength 488 nm and the emission was collected in the green light signal range. The fluorescence intensity was quantified by a CellQuest software (BD Biosciences).

### Western blot

The cells were plated at a density of 1 × 10^6^ cells per 60-mm Petri dish in complete medium for 16–20 h. Then the cells were treated with ND-conjugates at 37 °C. At the end of treatment, the cells were lysed in the ice-cold cell extract buffer (pH 7.6) containing 0.5 mM DTT, 0.2 mM EDTA, 20 mM HEPES, 2.5 mM MgCl_2_, 75 mM NaCl, 0.1 mM Na_3_VO_4_, 50 mM NaF, 0.1% Triton X-100. The protease inhibitors including 1 μg/mL aprotinin, 0.5 μg/mL leupeptin, and 100 μg/mL 4-(2-aminoethyl) benzenesulfonyl fluoride were added to the cell suspension. The lysate was vibrated for 30 min at 4 °C and centrifuged at 10,000 rpm for 10 min. The protein concentrations were determined by the BCA protein assay kit (Pierce, Rockford, IL). The protein samples were separated on 8–12% sodium dodecyl sulfatepolyacrylamide gels and electrophoretic transfer of proteins onto polyvinylidene difluoride (PVDF) membranes. The PVDF membranes were blocked overnight at 4 °C using blocking buffer that contained 5% non-fat dried milk in solution with 50 mM Tris/HCl (pH 8.0), 2 mM CaCl_2_, 80 mM sodium chloride, 0.05% Tween 20 and 0.02% sodium azide. The membranes were sequentially hybridized with primary antibody and followed with a horseradish peroxidase-conjugated secondary antibody. The protein bands were visualized with an enhanced chemiluminescence assay (SuperSignal™ West Pico Chemiluminescent Substrate; Life Technologies). The gel digitizing software, Un-Scan-It gel (Ver. 6.1, Silk Scientific, Inc., Orem, UT), was used for semi-qualification.

### Immunofluorescence staining and confocal microscopy

The cells were cultured on coverslips, which were kept in 35-mm Petri dish at a density of 5 × 10^5^ per well for 16–20 h. After treatment with or without ND-conjugates, the cells were washed with PBS. The cells were fixed with 4% paraformaldehyde solution overnight at 4 °C. Then the cells were washed three times with PBS and non-specific binding sites were blocked in PBS containing 10% FBS and 0.3% Triton X-100 for 1 h at 37 °C. Thereafter, the cells were separately incubated with rabbit anti- p-H3S10 (1:100) antibody in PBS containing 10% FBS overnight at 4 °C, and washed three times with 0.3% Triton X-100 in PBS. Then the cells were incubated with anti-rabbit IgG-Hylite 488 (1:100) in PBS containing 10% FBS for 1 h at 37 °C, and washed three times with 0.3% Triton X-100 in PBS. The β-tubulin and nuclei were stained with the Cy3-labeled anti-β-tubulin (1:100) and Hoechst 33258 (Sigma Chemical Co., St. Louis, Mo), respectively. The samples were examined under a Multiphoton Confocal Microscope System (TCS-SP5-X AOBS, Leica, Germany).

### Mitotic index analysis

The mitotic arrest cells following treatment with or without ND-conjugates were counted by mitotic index. The adherent cells were cultured on coverslips in a 60-mm Petri dish for 16–20 h before treatment. After treatment, the cells were carefully and gently washed with PBS (pH 7.4) to avoid the loss of mitotically round-up cells, and then fixed with 4% paraformaldehyde solution in PBS for one hour at 37 °C. The β-tubulin was stained with the Cy3-labeled mouse anti-β-tubulin (1:50) for 30 min at 37 °C. The nuclei were stained with 2.5 μg/mL Hoechst 33258 for 30 min. The mitotic cells showed round-up morphology, compact chromosomes, spindle formation, and contained a complete cell membrane but did not produce the cell membrane blebbing or the formation of apoptotic bodies. Mitotic index was determined by the percentage of mitotic cell number/total counted cells.

### Sub-G1 fraction analysis

The cells were plated at a density of 7 × 10^5^ cells per 60-mm Petri dish in complete medium for 16–20 h. After drug treatment, the cells were collected and fixed with ice-cold 70% ethanol overnight at −20 °C. Thereafter, the cell pellets were treated with 4 μg/mL PI solution, which contained 1% Triton X-100 and 100 μg/mL RNase for 30 min. To avoid cell aggregation, the samples were filtered through a nylon mesh membrane. A minimum of ten thousand cells in each sample was analyzed by CellQuest software in flow cytometer (FACSCalibur, BD Biosciences). The percentage of sub-G1 fractions was quantified by ModFit LT software (Ver. 2.0 and Ver. 3.2, BD Biosciences).

### Annexin V-PI staining

The cells were plated in 60-mm Petri dish at a density of 7 × 10^5^ cells/well for 16–20 h. Then the cells were treated with or without ND-conjugates in complete medium. Thereafter, the cells were collected and suspended in 1x binding buffer and then added 5 μl of Annexin V-FITC and 50 μg/mL PI. The samples were incubated at room temperature for 5 min in the dark and analyzed by flow cytometer (FACSCalibur, BD Biosciences). The cells shows Annexin V^+^/PI^−^ indicated at the early stage apoptosis. The Annexin V^+^/PI^+^ indicated the late stage apoptosis. The percentage of Annexin V-PI staining cells was quantified from a minimum of ten thousand cells by CellQuest software (BD Biosciences).

### Time-lapse observation of apoptosis induction

To observe the real-time formation of mitotic catastrophe and apoptosis by ND-conjugates, the cells were treated with ND-conjugates and then immediately observed by a live cell imaging microscope system for 24 h record (OLYMPUS IX71, Japan). The pictures and videos were edited by DP manager software (Ver. 3.3.1, OLYMPUS).

### Xenograft human CRC tumors in nude mice

The BALB/cAnN.Cg-*Foxn1* ^*nu*^/CrlNarl mice (4-week-old male) were obtained from the Laboratory Animal Center of NAR Labs (NAR Labs, Taipei, Taiwan). After a week for environmental adaption, the mice were established solid human colon tumors by subcutaneous injection of 2 × 10^6^ RKO cells. After 10 days, the mice bearing RKO xenografted tumors were treated with vehicle (PBS) or 50 mg/kg of ND-PTX by every 3 days for three times. The growth ability of tumors in the mice was measured by a digital caliper every three days and calculated by the following formula: (length) × (width)^2^ × 0.5. The tumors volumes were continuously measured until the mice were sacrificed. The Committee of Animal Center of National Chiao Tung University approved all animal studies and experimental protocols. All the methods involving animals were carried out in accordance with the approved guidelines.

### Time-lapse observation of human CRC tumors in nude mice

The HCT116-luc2 cell line was purchased from PerkinsElmer Inc. (Waltham, MA). The BALB/cAnN.Cg-*Foxn1* ^*nu*^/CrlNarl mice (4 to 6 week-old male) were obtained from the Laboratory Animal Center of NAR Labs (NAR Labs, Taipei, Taiwan). The Solid HCT116-luc2 flank tumors in mice were established by subcutaneous injection of 2 × 10^6^ cells. After 7 to 10 days inoculation, the mice bearing xenografted colorectal tumors were received 20 mg/kg of Cet, ND-PTX or ND-PTX-Cet. For the assessment of drug delivery in nude mice, the mice were anesthetized with 2% isoflurane and inoculated 100 μL 50 mM luciferin as substrate to emit luminescence and observed by IVIS spectrum imaging system (Xenogen IVIS Spectrum, PerkinElmer, Waltham, MA). Photographic and luminescence images were taken at constant exposure time. The acquired images and the luminescence intensity of harvested colorectal tumors were analyzed by Living Image® (Caliper Life Science).

### Tissue homogenizing assays

The harvested tumor tissues from sacrificed mice were collected in the ice-cold cell extract buffer (pH 7.6), which contained 0.5 mM DTT, 0.2 mM EDTA, 20 mM HEPES, 2.5 mM MgCl_2_, 75 mM NaCl, 0.1 mM Na_3_VO_4_, 50 mM NaF, 0.1% Triton X-100. The total cellular protein extracts were prepared. The protease inhibitors including 1 μg/mL aprotinin, 0.5 μg/mL leupeptin, and 100 μg/mL 4-(2-aminoethyl) benzenesulfonyl fluoride were added to the samples. The samples were homogenized by homogenizer (Model Pro 200, PRO Scientific Inc., Oxford, CT). The lysates were centrifuged at 10,000 rpm at 4 °C for 10 min. The protein concentrations were determined by the BCA protein assay kit (Pierce, Rockford, IL). The samples were further subjected to western blot analysis.

### Immunohistochemistry

Immunohistochemical staining was performed at the Pathology Core Laboratory, National Health Research Institutes in Taiwan. The tissue sections in slides were treated by xyline for deparaffinization and followed by 95 °C antigen retrieval in commercial Tris-base buffer with boric acid and ethylene-diamine- tetraacetic acid (Cell Conditioning 1, Roche, Rotkreuz, Switzerland) for 30 minutes. Then the samples were incubated with primary antibody for phospho-H3S10 with a dilution of 1:50 and Caspase 3 with a dilution of 1:200 overnight at room temperature. The detection was performed by the Ventana Discovery XT staining system with ultraView Universal DAB detection kit (Ventana Medical Systems, Inc, Tucson, AZ) according to the manufacturer’s protocol.

### Statistical analysis

Each experiment was repeated at least three times. Data were analyzed using Student’s *t-*test or analysis of variance (a comparison of multiple groups), and a *p* value of <0.05 was considered statistically significant in each experiment.

## Electronic supplementary material

Supporting figures

Supporting video
