# Supplementary material for: Co-delivery of paclitaxel and cetuximab by nanodiamond enhances mitotic catastrophe and tumor inhibition
Source: Sci Rep. 2017 Aug 29;7:9814. doi: 10.1038/s41598-017-09983-8 (PMC5575327; doi:10.1038/s41598-017-09983-8)
Supplement: Supplementary file 1 — Supporting figures [file 41598_2017_9983_MOESM1_ESM.pdf]

Co-delivery of paclitaxel and cetuximab by nanodiamond enhances mitotic catastrophe and tumor inhibition

Yu-Wei Lin[1,2,#], Emmanuel Naveen Raj[2,#], Wei-Siang Liao[2,#], Johnson Lin[3,#], Kuang-Kai Liu[2], Ting-Hua Chen[1], Hsiao-Chun Cheng[2], Chi-Ching Wang[2], Lily Yi Li[4], Chinpiao Chen[5] and Jui-I Chao[1,2,*]

[1]Department and Institute of Biological Science and Technology, National Chiao Tung University, Hsinchu 30068, Taiwan

[2]Institute of Molecular Medicine and Bioengineering, National Chiao Tung University, Hsinchu 30068, Taiwan

[3]Hemato-Oncology Section, Department of Internal Medicine, Mackay Memorial Hospital, Taipei 10449, Taiwan

[4]Department of Pharmaceutical Science, University of Toronto, Toronto, Ontario M5S 3M2, Canada,

[5]Department of Chemistry, National Dong Hwa University, Hualien 97401, Taiwan

[#]Equal contribution in this study

*To whom correspondence should be addressed to: Department and Institute of Biological Science and Technology, National Chiao Tung University, 75, Bo-Ai Street, Hsinchu 30068, Taiwan. Fax: 886-3-5556219. E-mail: jichao@faculty.nctu.edu.tw

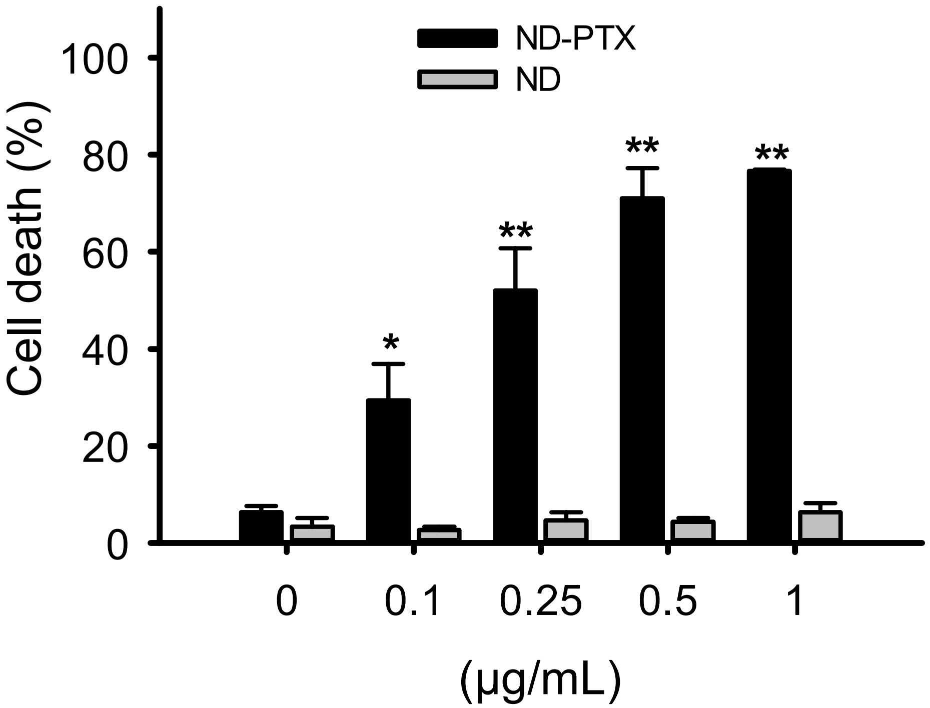

Figure S1 | ND-PTX induced cell death but not NDs in RKO cells. RKO cells were treated with ND or ND-PTX (0.1-1 μg/mL for 48 h). The percentage of cell death was determined by automatic cell-death counter. The above results were obtained from three separate experiments. The bars represent mean ± S.E. *$p < 0.05$, and **$p < 0.01$ indicate significant difference between control and ND-PTX treated samples.

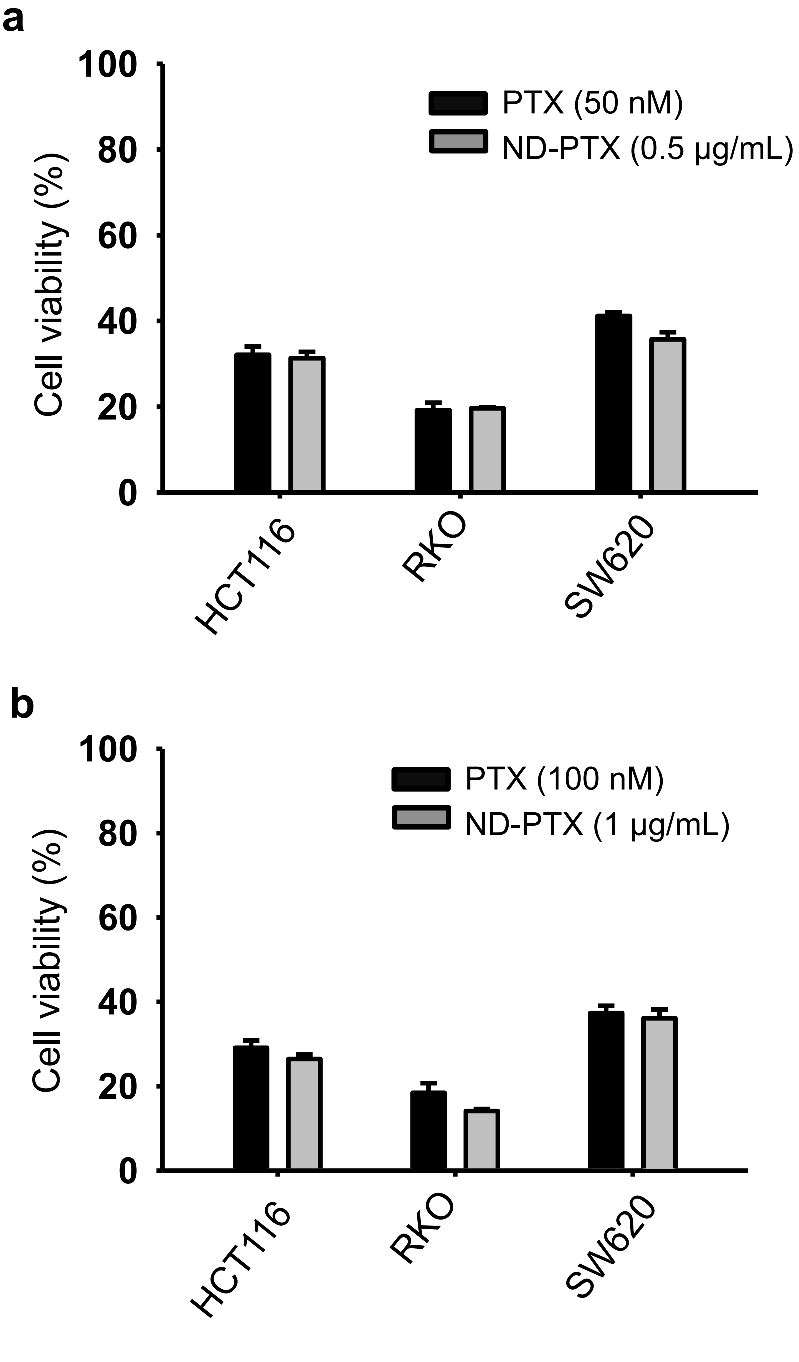

Figure S2 | Comparison of cell viability of ND-PTX and PTX in various CRC cell lines. (a) The HCT116, SW620 and RKO cells were separately treated with PTX (50 nM for 48 h) or ND-PTX (0.5 μg/mL for 48 h). (b) The above cells were treated with PTX (100 nM for 48 h) or ND-PTX (1 μg/mL for 48 h). At the end of treatment, the cell viability was determined by MTT assays. The above results were obtained from three separate experiments. The bars represent mean ± S.E.

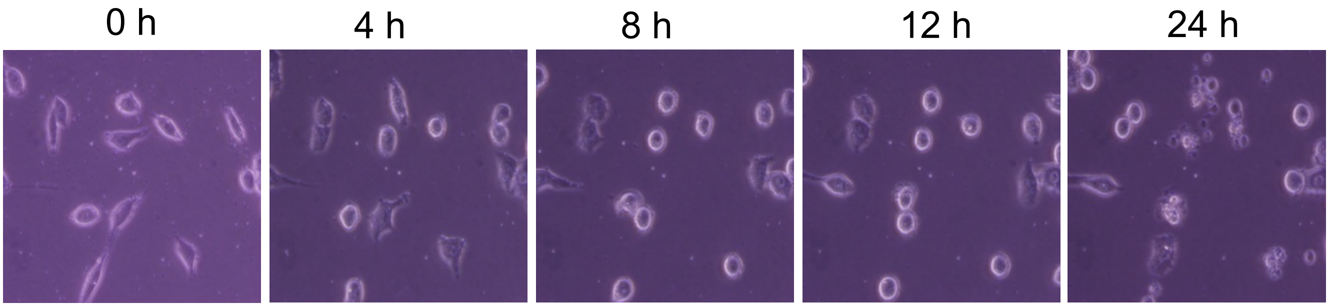

Figure S3 | ND-PTX induced cell death in CRC cells. RKO cells were treated with ND-PTX (1 µg/mL) and then recorded for 24 h. The cells were observed by a live-cell imaging microscope for time-lapse recording.

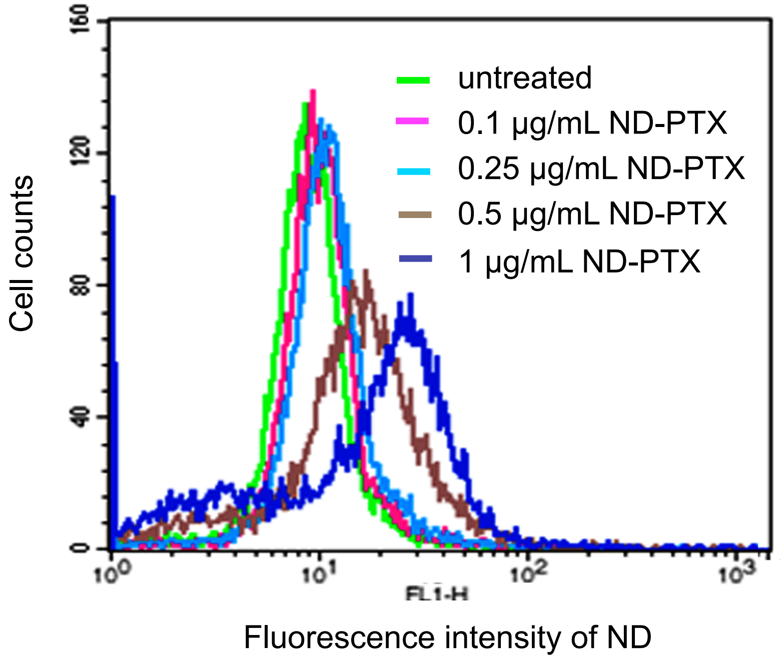

Figure S4 | ND-PTX increased cellular uptake into CRC cells in a concentration-dependent manner. RKO cells were left treated with or without 0.1-1 μg/mL ND-PTX for 48 h. The uptake ability of ND-PTX in CRC cells was analyzed by flow cytometer.
